# Persistent High-Risk HPV Infection and Molecular Changes Related to the Development of Cervical Cancer

**DOI:** 10.1155/2020/6806857

**Published:** 2020-07-23

**Authors:** Pablo Moreno-Acosta, Alfredo Romero-Rojas, Nicolas Vial, Antonio Huertas, Jinneth Acosta, Diana Mayorga, Schyrly Carrillo, Monica Molano, Oscar Gamboa, Martha Cotes, Camila Casadiego, Alexis Vallard, Nicolas Magne

**Affiliations:** ^1^Research Group in Radiobiology Clinical, Molecular and Cellular, National Cancer Institute, Bogota, Colombia; ^2^Research Group in Cancer Biology, National Cancer Institute, Bogota, Colombia; ^3^Group of Pathology Oncology, National Cancer Institute, Bogota, Colombia; ^4^Department of Radiation Oncology, Institut de cancérologie de la Loire-Lucien Neuwirth, 108 bis, Avenue Albert Raimond, BP 60008, 42271 Saint-Priest en Jarez, France; ^5^Biobank, Group of Pathology Oncology, National Cancer Institute, Bogota, Colombia; ^6^Pathology Group, National University of Colombia, Bogota, Colombia; ^7^Microbiology and Infection Diseases, The Royal Women's Hospital, Melbourne, Australia; ^8^Unit of Analysis, National Cancer Institute, Bogota, Colombia; ^9^Group Area Radiotherapy Oncology, National Cancer Institute, Bogota, Colombia

## Abstract

This article is a preliminary investigational study that is aimed at giving hints about the interesting biomarkers involved in the transition process from low-grade cervix lesion to invasive cervical cancer. Our study focuses on the risk factors and tumour molecular changes in one patient. First in 1986, she was diagnosed a preinvasive cervix lesion. Then, 16 years later, she was diagnosed an invasive cervical cancer. The 2002 diagnosis was a squamous cell carcinoma of the cervix, stage IIIB (FIGO), whereas in 1986, she had been diagnosed a high-grade squamous intraepithelial cervical lesion. Retrospectively, the analysis of samples of preneoplastic lesions and invasive cervical cancer confirmed the histopathological diagnoses and detected the presence of HPV type and HPV-16 variants, as well as the overexpression of proteins such as hTERT, IGF1R*α*, IGF1R*β*, CAIX, and GLUT1. Finally, the Arg72Pro polymorphism was detected in TP53. The role of high-risk HPV and HPV-16 variants and of hTERT, IGF1R*α*, IGF1R*β*, CAIX, and GLUT1 variations seemed confirmed in the development and progression of cervical cancer. As a result, analyzing the molecular changes in one and same tumour that progresses from a low-grade cervix lesion to invasive cervical cancer could provide valuable information in order to improve detection, diagnosis, and treatment in the future.

## 1. Introduction

According to ICO/IARC Information Centre about human papillomavirus (HPV) and cervical cancer (CC), in 2018, in Colombia, 18.75 million women were at risk of developing cervical cancer (aged over 15). Cervical cancer was the third most frequent cancer and the third most frequent cancer in women aged between 15 and 44. Every year, cervical cancer is diagnosed in 3853 Colombian women, and it claims the lives of 1775. Approximately 4.5% of women are infected with HPV-16 and HPV-18 at some point in their lives [1]. CC begins with the appearance of preneoplastic lesions. They result from HPV infection in cells located in the transformation zone of the cervical epithelial tissue [2, 3]. Low-grade or high-grade lesions appear. Over time, they form carcinoma in situ [2–5] and for unknown reasons, a significant proportion of lesions tend to turn into invasive cancer [4–6]. HPV infections are transient and most of the time cleared within a couple of years following exposure [7]. However, 10–20% of infections persist latently, leading to disease progression and, ultimately, various forms of invasive cancer [7]. Sexual behavior, age at first sexual intercourse, and the number of sexual partners are HPV risk factors [8, 9]. But, cervical cancer is not provoked by HPV infection alone. Immunosuppression, high parity, long-term use of oral contraceptives, cigarette smoke, HIV status, herpes and chlamydia genital infections, age, race, family history of cervical cancer, difficulty to have access to gynecologic follow-up, and overweight also increase the risk of CC [8, 9].

The neoplastic progression or transformation of preneoplastic lesions in invasive cancer cells is provoked at least partly by HPV, especially through gene and protein expression modulation [2, 5, 6, 10]. This modulation involves oncogene activation, loss of tumor suppressor genes (mostly p53) and alterations in molecular signaling pathways. Protein expression modulation can be considered as a promising hypothetical model. It may refer to characteristic of the tumor microenvironment, such as hypoxia (low oxygen levels up to 1%), increased glycolysis, and acidosis (low pH) [10–13]. Indeed, the malignant cells use alternative metabolic pathways to develop and survive [12, 13]. For several years, we studied the modulation of genes and proteins such as IGF1R, IGF-I, GF-II, GAPDH, HIF-1 alfa, Survivin, GLUT1, CAIX, HKII, and hTERT (status methylation and protein expression) and the presence of HPV in cervical cancer and response to treatment. So did we with the methylation status of hTERT and hTERT protein expression. The results of our studies showed that response to treatment, gene expression, and protein expression in the presence of high-risk HPV and variants of HPV-16 were linked. Through these studies, we contributed to the identification of predictive and prognostic biomarkers [10–15]. Recent analyses have aimed at improving the assessment of patients' individual risks [16–18]. The biomarkers we analyzed could be related to the development of preneoplastic lesions in women at risk of having cervical cancer. As a result, routine surveillance of such biomarkers could lead to cost-effective detection in developing countries [1, 4, 8, 9]. Yet, in such countries, the health system does not include biomarkers detection. Thus, the molecular analysis of one and same tumour evolving from low-grade to invasive cervical cancer could improve current responses to prevention, diagnosis, and treatment [18, 19]. The present study focuses on the molecular analysis of a 16-year disease progression from preinvasive cervix lesion to invasive cervical cancer in one patient.

## 2. Case Presentation

**Figure 1:**
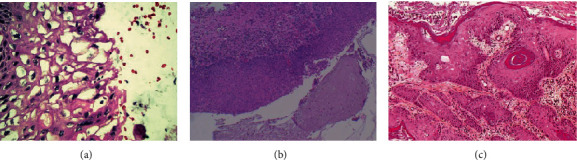
Key histological images of preneoplastic lesions and invasive cervical cancer. (a) CIN I associated to HPV infection (koilocytes). (b) CIN II-III associated to HPV infection (koilocytes). (c) Invasive squamous cell carcinoma.

**Figure 2:**
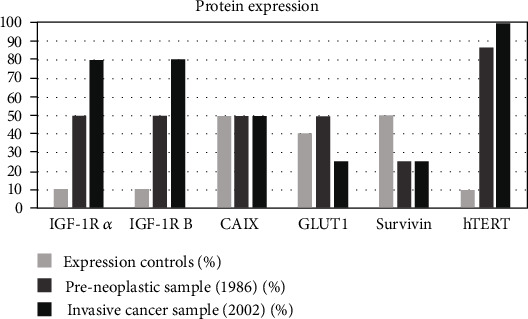
Protein expressions in preneoplastic lesions, invasive cancer, and controls.

**Figure 3:**
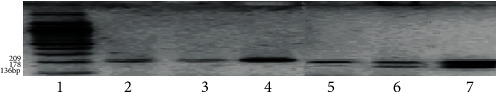
Allele-specific PCR-Arg72Pro. Lanes: 1: molecular weight marker; 2-4: *β*-globina amplicon 209 bp: S1 (paraffin block sample—1986), S2 (paraffin block sample—2002), H (HeLa); 5-7: Arg72Pro P53 amplicons (178 bp Pro, 136 bp Arg): S1, S2, and H (HeLa: heterozygous genotype Arg72Pro).

The patient was referred for diagnosis and management to the National Cancer Institute in 1986. Her high-grade squamous intraepithelial lesion was identified during routine Pap tests. Gynecologic history was as follows: she was 13 when menarche, first sexual intercourse when 15, first childbirth when 16, and finally had seven other children. She had a single sexual partner. Colposcopy revealed ectopy 1, acetowhite epithelium, mosaic, type 3 vessels, and acetowhite endocervical epithelium with papillary excrescences. Two biopsies confirmed the diagnosis of high-grade squamous intraepithelial lesion CIN II-III with glandular extension. The second biopsy also identified CIN of low-grade I-HPV squamous intraepithelial lesion. An extended hysterectomy was recommended by the medical oncology board. Yet, the patient refused and only returned to the institution in 2002 due to uterine bleeding. The colposcopy showed an exophytic mass of more than 5 cm that replaced the cervix, and the biopsy confirmed the diagnosis of invasive carcinoma, stage IIIB in the FIGO classification. The patient finally died in 2005, after voluntarily canceling the planned curative radiotherapy. The histological slides of the samples from 1986 to 2002 were retrospectively reviewed and two onco-pathologists confirmed the diagnoses. In the 1986 slides, they identified a high-grade squamous intraepithelial lesion—CIN II-III—with glandular extension (Figure 1(b)) and low-grade squamous intraepithelial lesion—CIN I, associated with HPV infection (Figure 1(a)). In the 2002 slides, invasive squamous cell carcinoma was confirmed as invasive carcinoma type large squamous cell carcinoma, nonkeratinizing, infiltrating, and moderately differentiated of the cervix (Figure 1(c)). The quality of paraffin that embedded tissue samples and frozen fresh tissue samples made PCR possible for a 209 bp fragment of the *β*-globin gene [20]. HPV and HPV variants were detected by means of PCR and sequencing and reverse line blot [20, 21]. Seventy different molecular variants of the HPV-16 E6 gene and its six major phylogenetic branches, European (E), Asian (As), Asian-American (AA), African-1 (Af1), African-2 (Af2), and North-American (NA1) [20, 22, 23], were searched for on the 1986 (paraffin-embedded tissue) and 2002 (fresh frozen tissue and paraffin-embedded tissue) samples. Multiple infections by HPV-16 and HPV-33 was detected in the 1986 sample, and HPV-16 simple infection was detected in the 2002 sample. Coinfection with HPV-16 with the presence of the Asian-American subclass c variant (AA-c) and the European variant HPV-16 350 (E-G350) was found both in the 1986 preneoplastic samples and the 2002 samples from invasive cancer. As to the viral load, the amplification signal by PCR was more intense for HPV-16 than for HPV-33. Immunohistochemistry (IHC) was performed to evaluate the expressions IGF1R*α*, IGF1R*β*, Survivin, GLUT1, CAIX, and hTERT specifically at the level of nuclear and cytoplasmic localization. Two oncopathologists performed the evaluation of each marker. The staining intensity score was defined as extension of positivity (percentage of positive cells: positive +: 11-50%; positive ++: 51-80%; positive +++: >80%). A negative expression was defined by a staining <10%. Rating scales were detailed elsewhere [11, 14]. Patients' biopsies in age group 30-60 with histological diagnosis of cervicitis but without intraepithelial lesion or malignancy were used as controls. The detection of protein expression compared to the obtained for controls showed an increase in the expression of IGF1R*α*, IGF1R*β*, and hTERT (expression >80% was observed in nucleus as cytoplasm) as well as a decrease in the expression of Survivin in preneoplastic samples and invasive cancer samples. The expression of CAIX and GLUT1was similar both in preneoplastic samples and in control group. GLUT1 expression in invasive cancer samples was lower than in the control group. Strong expressions of CAIX, GLUT1, and hTERT in preneoplastic lesions were observed while a weak expression of GLUT1 and Survivin was observed in cancer samples (Figure 2). TP53 arginine72proline polymorphism was analyzed by allele-specific PCR [24]; HeLa cell line presenting heterozygosity for the polymorphism of the TP53 gene was used as a positive control. The study of the TP53 polymorphism revealed an arginine/proline genotype in both samples (from the preinvasive in 1986 and the invasive cancer in 2002). The amplification signal by PCR was more intense for proline than for arginine (Figure 3).

## 3. Discussion

The patient had well-known risk factors to develop persistent HPV infection and cervical cancer, namely, grand multiparity, early age of first intercourse, and first childbirth. Molecular change (between 1986 and 2002) analysis permitted to detect the presence and type of HPV and HPV16 variants, as well as about the overexpression of proteins such as hTERT, IGF1R*α*, IGF1R*β*, CAIX, and GLUT1. Finally, the Arg72Pro polymorphism was detected in TP53. The role of high-risk HPV and HPV-16 variants of the variation of hTERT, IGF1R*α*, IGF1R*β*, CAIX, and GLUT1 is suggested in the development and progression of cervical cancer. As a result, analyzing the molecular changes in one and same tumour that progressed from a low-grade stage to invasive cervical cancer could provide valuable information in order to improve detection, diagnosis, and treatment in the future.

Possible modulators of oncogenesis were identified on precancerous samples, with a HPV-16 coinfection and a TP53 polymorphism [25, 26]. Interestingly, these elements were also identified on invasive cancer samples 16 years later. HPV-16 persistence after therapy was associated with poorer outcomes, more precisely with increased local recurrences [27–29]. HPV persistence is thought to be caused by the intervention of HPV on coproteins at the cell cycle level and to take part in the immortalization of keratinocytes [7, 30], leading tissues with CIN III to cervical cancer [7, 28–30]. Several HPV-16 variants have been identified in literature, with six major branches: European (E), Asian (As), Asian-American (AA), African-1 (Af1), African-2 (Af2), and North-American (NA1). It was previously suggested that HPV variants might differently impact the whole oncogenic process, including the virus assembly, the immune recognition, the p53 degradation, and finally, the processes of cell immortalization [22, 23]. Regarding the HPV variants and especially that of the HPV-16 variants in the development and progression of cervical cancer, recent studies have contributed to a better understanding of the role they play [27, 30]. In the association of the variant E-T350G with the development of cervical cancer, opposite findings are observed due to regional heterogeneity. In Europe, although with few differences, 350T infections are more likely to persist and progress to CIN-III than in South and Central America, where the 350G variant is strongly associated with cervical cancer [30]. The present case showed a patient with CIN II-III and presence of coinfection by HPV-16 AA-c, HPV-16 E-G350, and HPV-33. Progression to invasive cancer was observed 16 years later.

Early detection programs for the prevention of cervical cancer in developing countries are based on the assessment of individual genotypes or the combination of different high-risk HPV genotypes [27]. In the present report, HPV-16 and HPV-33 coinfection was detected in preneoplastic lesion samples. Coinfection was not subsequently detected in invasive cancer samples. It indicated that HPV-33 infection was eliminated. This suggests that persistent infection by HPV-16 variants as well as viral load and age could be necessary factors for the progression of the disease. In addition, the detection of specific variants such as E-G350 and AA-c of HPV-16 reported in this case could help to identify patients with a high risk of viral persistence and development of cervical neoplasia [27, 30].

The TP53 gene promotes the development of cervical cancer. The TP53 (product of the TP53 gene) acts as a tumor suppressor. It arrests the cell cycle in G1 so that DNA damage can be repaired before DNA replication [5, 6]. The relationship between the TP53 Arg72Pro polymorphism and cervical cancer is probably modulated by the presence of high-risk HPV during progression from preinvasive lesions through to cervical cancer [25, 26]. Habbous et al. reported that the most common P53 genotype in HPV-associated tumors was homozygosis arginine/arginine [26]. Authors evidenced that this homozygosis was a risk marker of developing cervix cancer, suggesting a high sensitivity of arginine to HPV E6-mediated degradation [25, 26]. However, several single-nucleotide polymorphisms were identified in the TP53 gene, probably inducing different sensitivities to HPV E6. The most frequent polymorphism is located on the exon 4 codon 72, with the replacement of arginine by proline, also named the “Arg72Pro” genotype. Variants containing proline were suggested to be less likely to be degraded by HPV E6 protein than by the protein containing arginine only [25, 26]. In the present case, the heterozygous genotype containing proline might explain the surprisingly long time (more than 16 years) between diagnosis of precancerous lesions and of cervical cancer without any treatment. Previously described anomalous molecular signaling pathways were identified in invasive cervical cancer samples, especially with strong expressions of IGF1R *α* and *β* [10]. IGF1R is a tyrosine-kinase receptor promoting mitogenic, metastatic, and antiapoptotic phenotypes in several cancers [10]. In cervical cancer, IGF1R was previously described as a predictive biomarker of tumor aggressiveness [31, 32] through hypoxia-mediated phenomenon [10, 13, 14]. The percentage of expression of IGFIR *α* and *β* was higher in both preneoplastic (1986) and invasive cancer (2002) than in controls. The increasing expression reported between preneoplastic and neoplastic tissues suggested that IGF1R might play an early and key role in invasive cancer development [31]. It promoted insidious cell proliferation and anomalous survival in preneoplastic tissues, with a fostered effect in neoplastic tissues [32]. Interestingly, it was suggested that HPV-16 interacted with IGF1R in cervical tumors, resulting in radio resistance [13, 14, 33, 34]. Moreover, Zacapala-Gómez et al. suggested that the expression of E6 variants AA-c and E-G350 induced overexpression of IGF1R [35]. Therefore, the persisting coinfection of HPV-16 reported in the present case might have directly promoted the reported dysregulation of IGF1R.

Strong expressions of hTERT, GLUT1, and CAIX in preneoplastic lesions were observed while a weak expression of GLUT1 and Survivin was observed in cancer samples. One of the key findings in this case study was the subcellular localization of hTERT. It showed that hTERT was expressed both in the nucleus and in the cytoplasm of preneoplastic lesions and cervical cancer. The expression of the hTERT protein was studied in chronic cervicitis, intraepithelial neoplasms, and invasive cervical cancer in different studies. An increase in hTERT expression as cervical disease progresses was evidenced [36]. This increase in hTERT expression may be due to the action of high-risk HPVs. Nonregular activities such as apoptosis blocking, gene transcription, and cell proliferation were modulated. This increase could have significant functions in the HPV viral life cycle; therefore, it could play an essential role in cell immortalization and in the malignant transformation process [37, 38]. Some studies reported that GLUT1 overexpression was related to the grade of the tumor, but not to the progression of preneoplastic lesions [39]. However, when compared to the control group, our case showed a slight increase in expression in preneoplastic lesions, and a decrease in invasive cancer was observed.

CAIX is an endogenous marker of hypoxia that is commonly overexpressed in cervical cancers [11, 12]. CAIX overexpression derives from the upregulation of HIF-1 alpha, a protein induced by hypoxia that upregulates prosurvival and proproliferation signaling pathways [11, 12]. Survivin was also previously related to hypoxic cervical cancer cells phenotypes [14, 40]. The present results support the hypothesis that these proteins could play a key role in the tumor phenotype establishment, mediated by hypoxia. The prevalence of HPV-16 variants; the presence of Arg72Pro3 polymorphism, IGF-1R, CAIX, GLUT1, Survivin, and hTERT expressions; and their relationship with cervical cancer development and recurrence were previously studied in cervical cancer patient undergoing radiation or chemoradiation. IGF1R overexpression and IGF1R detection by IHC were associated with a poor prognosis. Therefore, they turned into possible interesting biomarkers of resistance of treatments [13, 14, 34]. Thus, analyses of biological and molecular changes (performed on preneoplastic lesions and locally advanced cancer) could be considered valuable biomarkers, reflecting the current or future aggressiveness of the disease. If current hypotheses are validated by additional studies, such an analysis of molecular changes could guide the selection of the most appropriate detection, diagnosis, and treatment [19, 41].

The retrospective study of this case permitted to describe the risk factors and molecular changes in one and same tumor at two periods during development and progression of the disease. As a retrospective study, it has limitations and strengths. On the one hand, the preexisting information collected, diagnostic procedures, such as screening tests for the diagnosis of injuries preinvasive and cervical cancer (conventional cytology used as part of the initial diagnosis), preservation of tissue in formalin and inclusion in paraffin, age of paraffin blocks, and quality of DNA are part of the limitations. On the other hand, we were able to observe and analyze an identified case of invasive cervical cancer with a 16-year progression and to analyze of the molecular changes in the same tumor from a low-grade stage to invasive cervical cancer to provide valuable information. It could improve future responses to prevention, diagnosis, and treatment in the future. Furthermore, the techniques used in the analysis of molecular changes were highly sensitive and specific.

## Abbreviations

CAIX: Carbonic anhydrase IX
CC: Cervical carcinoma
CIN: Cervical intraepithelial neoplasia
DNA: Desoxyribonucleic acid
GLUT1: Glucose transporter 1
HIF: Hypoxia-inducible factor
HPV: Human papilloma-virus
hTERT: Human telomerase reverse transcriptase
IGF-1R: Insulin growth factor receptor 1
PCR: Polymerase chain reaction.
